# Decreased retinal thickness in patients with Alzheimer’s disease is correlated with disease severity

**DOI:** 10.1371/journal.pone.0224180

**Published:** 2019-11-05

**Authors:** Jae-Il Kim, Bong-Hui Kang

**Affiliations:** Department of Neurology, Dankook University College of Medicine, Dankook University Hospital, Cheonan, Korea; Niigata University, JAPAN

## Abstract

**Background and purpose:**

The loss of retinal ganglion cells observed in Alzheimer’s disease (AD) may be attributable to a neurodegeneration of the neuro-retinal structure. Amnestic mild cognitive impairment (aMCI) has been considered a prodromal stage of AD. We evaluated retinal thicknesses in patients with aMCI and AD compared to healthy controls using spectral-domain optical coherence tomography (OCT) to investigate whether changes in retinal thickness are correlated with the clinical severity of dementia.

**Methods:**

Patients with aMCI (n = 14), mild to moderate AD (n = 7), severe AD (n = 9), and age-matched controls (n = 17) underwent neuro-ophthalmologic examinations. Global deterioration scale (GDS), clinical dementia rating (CDR), and mini-mental status examination (MMSE) were used to evaluate the clinical overall severity of dementia. The thicknesses of the peripapillary retinal nerve fiber layer (RNFL), total macula, and macular ganglion cell-inner plexiform layer (GC-IPL) were measured using Cirrus HD-OCT.

**Results:**

The severe AD group had overall significantly thinner GC-IPL, total macula, and peripapillary RNFL compared to the controls (p<0.05). In the mild to moderate AD group, the total macula, average RNFL, and superior RNFL thickness were each significantly reduced compared to controls (p<0.05). The aMCI group had reduced total macula, average RNFL, and inferior RNFL thickness, but there were no significant differences compared to the controls. The GDS and CDR scores had a negative correlation with the thickness of the GC-IPL and the total macula. The MMSE scores had a positive correlation with both the total macular and average RNFL thickness, when adjusted for age (p<0.05).

**Conclusions:**

This study confirmed that retinal thickness is decreased in AD patients. There is a correlation between reduced retinal thickness and the clinical severity of dementia.

## Introduction

Alzheimer’s disease (AD) is the most common neurodegenerative dementia and is characterized by a progressive cognitive decline and impaired function in daily activities. Neuronal loss, neurofibrillary tangles, amyloid plaques, and granulovacuolar degeneration are the typical histopathologic features of AD in the brain, especially in the hippocampus and temporo-parietal cortex. These histopathologic findings are also observed in the retina of AD patients, including amyloid plaques [1], retinal ganglion cell loss [2], and optic neuropathy [3]. In postmortem histopathological studies, the main alterations were found in the macular area [2].

Mild cognitive impairment (MCI) is defined as impairment in cognitive functions with otherwise normal performance of daily living activities; it is considered a transitional stage between normal aging and dementia [4]. When memory loss is the predominant symptom, it is termed amnestic MCI (aMCI) and is frequently seen as a prodromal stage of AD [4, 5]. Studies have suggested that aMCI patients progress to probable AD at a higher rate (approximately 10% to 15% per year) than normal controls (1–2% per year). Furthermore, 80% of these aMCI patients were later diagnosed with AD after approximately 6 years of follow-up [4].

Optical coherence tomography (OCT) is a non-invasive, non-contact, trans-pupillary imaging technology that provides high-resolution cross-sectional images of the retinal nerve fiber layer (RNFL) and measures macular thickness and volume. More recent advances in segmentation algorithms have made it possible to use OCT to visualize and measure individual retinal layers in the macular region. The advancement of OCT has enabled the assessment of the retinal ganglion cell complex in the macular area, which is the sum of the three innermost retinal layers: the RNFL, which is composed of axons; the ganglion cell layer (GCL), which is composed of cell bodies; and the inner-plexiform layer (IPL), which contains the retinal ganglion cell dendrites as well as the peripapillary RNFL thickness. Considering that the retina is essentially an extension of the brain and is a part of the central nervous system, OCT has the potential to become a non-invasive and reproducible test for axonal degeneration of the retina, which can be a potential surrogate marker for neurodegeneration [6].

Recent studies have shown that the peripapillary RNFL thickness is reduced in AD patients compared with healthy individuals [7, 8]. However, the few studies which included MCI patients in their evaluations had inconsistent conclusions [9–12]. Additionally, it is uncertain whether retinal degeneration occurs in parallel with dementia progression. Therefore, we evaluated retinal thickness in patients with aMCI and AD using spectral-domain OCT (SD-OCT) to investigate whether changes in retinal thickness are correlated with the clinical severity of dementia.

## Methods

### Subjects

We performed a cross-sectional study, calculating sample sizes from previous reports and our preliminary study [13] based on difference values and standard deviation (SD) of RNFL thickness between AD patients and controls. The expected sample size was about 30 subjects per group.

Patients were included in the study if they had a diagnosis of probable AD or aMCI according to clinical diagnostic criteria with comprehensive neuropsychological tests addressing five cognitive domains and brain magnetic resonance imaging. AD patients fulfilled the NINDS-ADRDA criteria [14] and the diagnosis of aMCI was based on the recommendations from the National Institute on Aging and the Alzheimer’s Association [5]. Cognitively healthy age-matched subjects were also included as controls. All subjects had detailed ophthalmologic examinations, including measurement of visual acuity, pupillary reflexes, slit-lamp examination, intraocular pressure by Goldmann applanation tonometry, gonioscopy, refraction test, Humphrey visual field test, and detailed fundus examination. We excluded subjects with known diseases affecting the eye or optic nerve, such as glaucoma, optic neuropathy, or diabetic retinopathy. In addition, eyes with unclear media (e.g., dense cataract) or with any pathological eye findings in the ophthalmic examinations that prevented OCT examination were excluded from the study.

The probable AD patients were divided into two groups based on their global deterioration scale (GDS). Patients in the mild to moderate AD group had a GDS score of 3 to 4. Patients in the severe AD group had a GDS score of 5 or greater. GDS, clinical dementia rating (CDR) and mini-mental status examination (MMSE) were used to evaluate the overall clinical severity of dementia [15]

Written informed consent was obtained from all subjects and the signed informed consent documents were kept on file. We received informed consent directly from control subjects and from patients with dementia, and their legal representatives. The protocol was approved by the Dankook University hospital ethics committee (IRB number: 2013-10-009) and it adhered to the tenets of the Declaration of Helsinki and its later amendments or comparable ethical standards.

### Optical coherence tomography

Cirrus HD-OCT software version 6.0.0.599 (Carl Zeiss) was used to acquire retinal thickness measurements [16]. Images were rejected if there were movement artifacts, segmentation errors or poor concentering on the fovea for the macular cube protocols.

**Fig 1:**
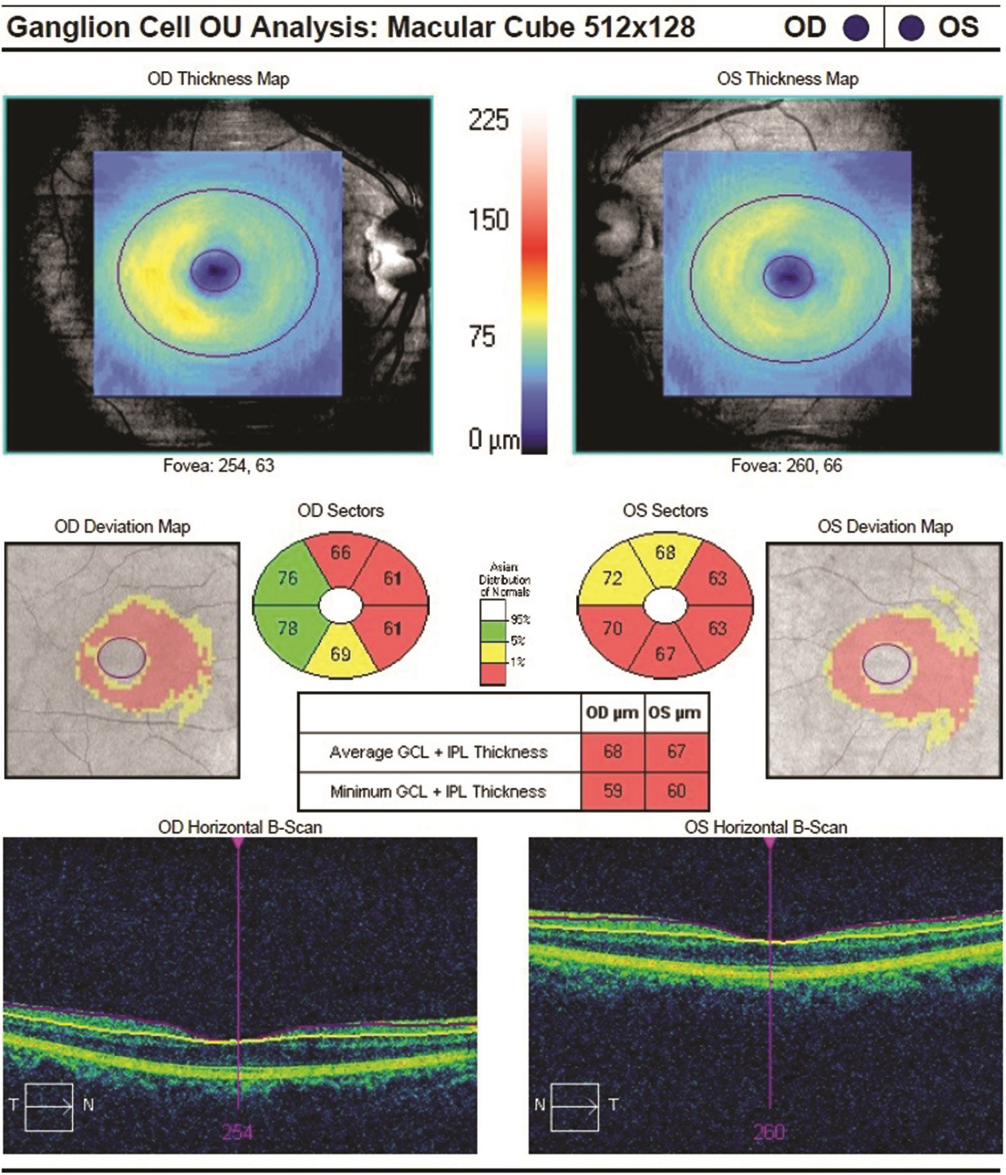
The Cirrus HD-OCT result of GC-IPL thickness in a patient with AD. OD: right eye, OS: left eye.

The Macular Cube 512 × 128 protocol was used for the mean macular and GCL plus IPL (GC-IPL) thickness measurements (Fig 1). The Cirrus HD-OCT ganglion cell analysis algorithm was used to process the data. It detected and measured the macular GC-IPL thickness within a 14.13-mm^2^ elliptical annulus area centered on the fovea and automatically identified the outer boundary of the RNFL and the IPL. The difference of RNFL and the IPL outer boundary segmentations yielded the combined thickness of the GC-IPL, which was indicative of the health of retinal ganglion cells in macular region. The average, minimum and sectoral thicknesses of the GC-IPL were measured in an elliptical annulus around the fovea.

**Fig 2:**
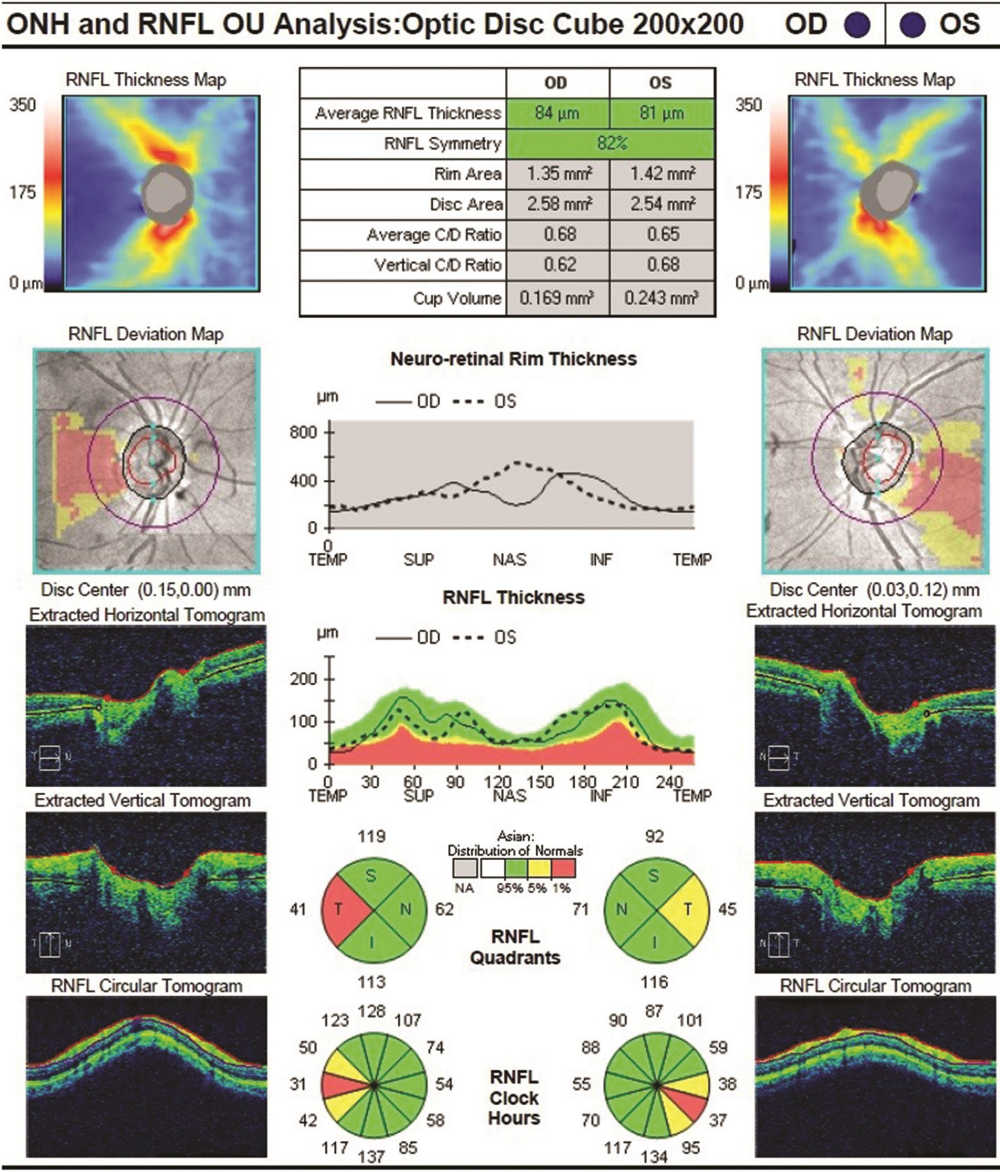
The Cirrus HD-OCT result of peripapillary RNFL thickness in a patient with AD. ONH: optic nerve head, OD: right eye, OS: left eye.

The Optic Disc Cube 200 × 200 protocol consisted of 40,000 axial scans in a 6 × 6 × 2 mm cube centered on the optic disc (Fig 2). Average RNFL thickness and RNFL value by quadrants (superior, inferior, temporal and nasal) and clock-hour sectors on a measurement circle 3.46 mm in diameter were calculated, and their deviation from a normative database was provided in a color-coded scheme.

### Statistical analysis

Statistical analyses were performed using Statistical Package for the Social Sciences for Windows version 23.0 (SPSS Inc., Chicago, IL, USA). A p-value <0.05 was considered statistically significant. Continuous data were reported as mean values ± SD. We used a mean of both eyes per patient for statistical analysis. If OCT results in only one eye were available, the monocular data were the representative values of the patient. The four groups of subjects were adjusted for age using the Kruskal-Wallis and Mann-Whitney tests, for gender using the chi-square test and for difference in OCT measurements using the analysis of covariance (ANCOVA) test. Standard multiple regression analysis was carried out to assess contributions of the clinical disease severity (the scores of MMSE, GDS and CDR) to OCT measurements (retinal thicknesses) while adjusting for age as a potential confounder.

## Results

### Demographics of the subjects (Table 1 and S1 File)

A total of 47 subjects were evaluated: 9 in the severe AD group, 7 in the mild to moderate AD group, 14 in the aMCI group, and 17 in the control group. There were no significant differences between the four groups regarding age and gender. There were significant differences in the MMSE, GDS, and CDR scores between the aMCI, mild to moderate AD and severe AD groups (p<0.001). The aMCI group had higher MMSE scores and lower GDS and CDR scores than both AD groups (p < 0.05). The severe AD group had lower MMSE scores and higher GDS and CDR scores than the mild to moderate AD group (p<0.05).

**Table 1:** Demographics of the four groups.

	Severe AD (N = 9)	Mild to moderate AD (N = 7)	aMCI (N = 14)	Control (N = 17)	P-value
**Age (years)**	74.2 ± 5.9	76.0 ± 11.1	68.6± 7.9	73.6± 7.5	0.107*
**Female: Male**	5:4	2 : 5	8 : 6	8 : 9	0.75**
**MMSE**	12.1 ± 5.1	18.4± 4.9	24.2± 2.0		**<0.001**^**¶**^
**CDR**	1.4 ± 0.7	0.9±0.2	0.5± 0.0		**<0.001**^**¶**^
**GDS**	5.3 ± 0.5	3.9± 0.4	3.0± 0.0		**<0.001**^**¶**^

N: number of subjects, MMSE: mini-mental status examination, CDR: clinical dementia rating, GDS: global deterioration scale,

* Kruskal-Wallis test,

** Chi-square test,

¶ ANCOVA (covariate: age),

**bold type** indicates statistical significance.

### Comparison of OCT measurements between the four groups (Table 2, Fig 3 and S1 File)

1. The severe AD group: The severe AD group had significantly thinner GC-IPL, total macula, and peripapillary RNFL than the controls (p<0.05). The RNFL thicknesses in the superior, inferior and temporal quadrants were significantly decreased when compared to the controls (p<0.05).
2. The mild to moderate AD group: The total macula, average RNFL, and inferior RNFL thickness in this group were each significantly lower than that of the controls (p<0.05).
3. The aMCI group: The aMCI group had reduced total macula, average RNFL, and inferior RNFL thickness, but there were no significant differences compared to the controls (p>0.05).

**Fig 3:**
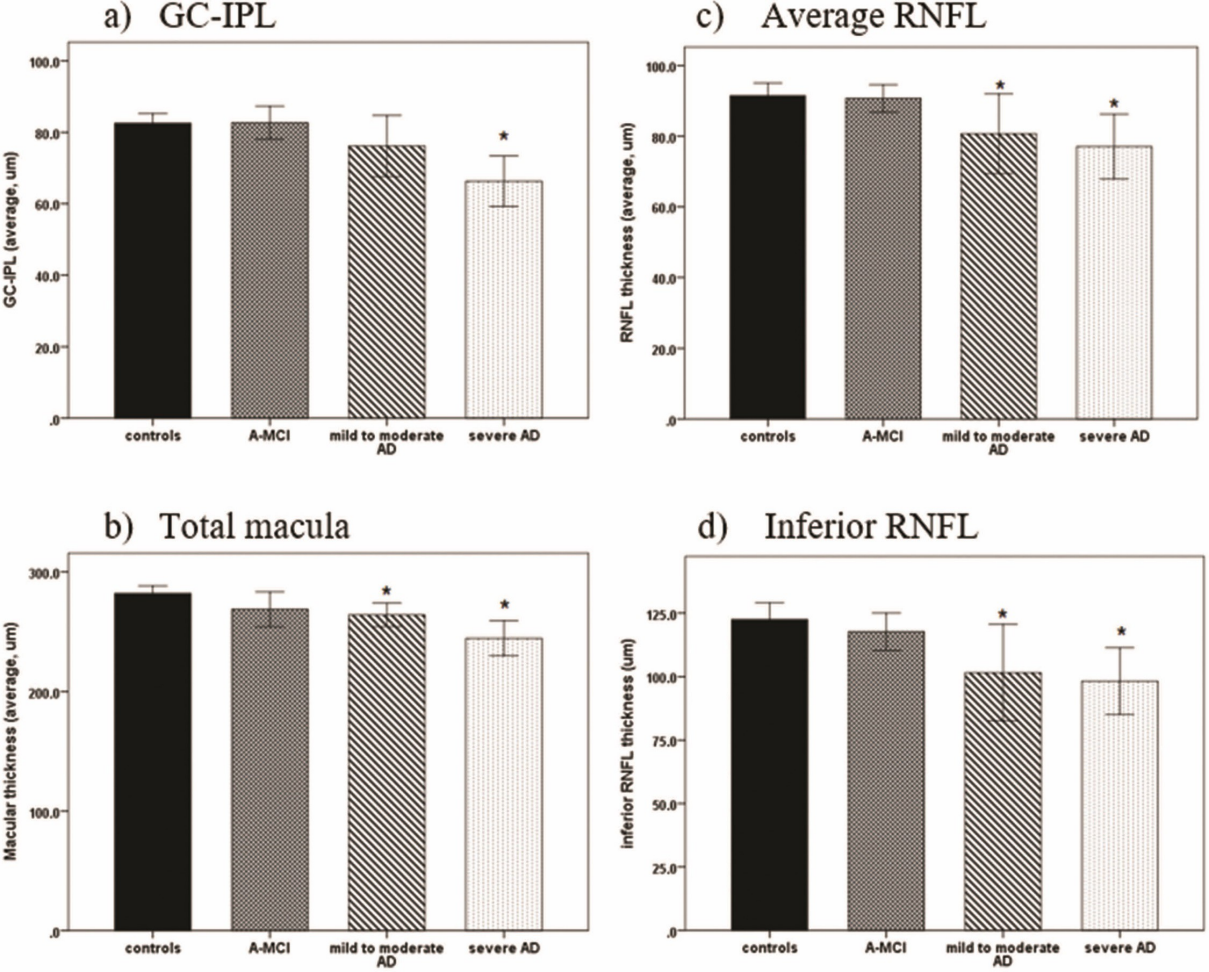
These diagrams represent the results of retinal thicknesses observed in the four groups. * Statistically significant difference compared with the controls (P<0.05).

**Table 2:** The GC-IPL, total macula, and RNFL thickness measurements in the four groups.

	Severe AD (N = 9)	Mild to moderate AD (N = 7)	aMCI (N = 14)	Control (N = 17)	P-value **
**Average GC-IPL**	66.3 ± 10.0*	76.2±10.5	82.7±8.0	82.5±5.6	**<0.001**
**Total Macula**	244.7 ± 20.6*	264.0±12.1*	268.7±26.3	282.1±12.6	**0.001**
**Average RNFL**	77.06 ± 13.0*	80.7±14.9*	90.7±7.3	91.5±7.4	**0.007**
**Inferior RNFL**	98.3 ± 18.6*	101.6±25.1*	117.8±3.7	122.4± 13.6	**0.004**
**Superior RNFL**	93.7 ± 19.5*	100.4±18.2	113.5±14.7	112.1±15.6	0.072
**Temporal RNFL**	56.1 ± 15.8 *	61.4±8.6	65.8±10.3	66.9±10.6	0.114
**Nasal RNFL**	60.0 ± 6.3	58.0±12.2	65.8±8.0	63.5±9.2	0.476

The results represent the mean (μm) ± SD values,

* P<0.05 vs controls,

** ANCOVA (covariate: age), **bold type** indicates statistical significance.

### The correlation between severity of dementia and retinal thickness (Table 3)

According to Table 3, the GDS, CDR and MMSE scores were significantly correlated to the OCT measurements when adjusted for age. If the GDS score increased by one, GC-IPL thickness was expected to decrease by 5.3 μm (ß = -5.3, p<0.001) and macular thickness was expected to decrease by 11.1 μm (ß = -11.1, p = 0.008). CDR scores also showed a negative correlation with the thicknesses of the GC-IPL (ß = -7.2, p = 0.031) and total macula (ß = -21.6, p = 0.005). MMSE scores had a positive correlation with the average RNFL thickness (ß = 1.12, p = 0.004) and total macula thickness (ß = 1.645, p = 0.021).

**Table 3:** Multiple regression analysis with OCT measurements as dependent variables to assess contributions of the clinical disease severity adjusted by age.

Severity of dementia	OCT measurements (dependent variables)	Regression coefficient ß	Standard error	R^2^	p-value
**GDS**	GC-IPL	-5.3	1.62	0.352	<0.001*
	Total macula	-11.1	3.82	0.252	0.008*
	Average RNFL	-0.28			0.104
**CDR**	GC-IPL	-7.2	3.12	0.373	0.031*
	Total macula	-21.6	6.91	0.272	0.005*
	Average RNFL	-9.0		0.357	0.076
**MMSE**	GC-IPL	0.31			0.124
	Total macula	1.645	0.67	0.194	0.021*
	Average RNFL	1.12	0.35	0.273	0.004*

OCT: optical coherence tomography, MMSE: mini-mental status examination, CDR: clinical dementia rating, GDS: global deterioration scale,

* bold type indicates statistical significance.

## Discussions

In this study, we evaluated the thickness of the peripapillary RNFL, total macula, and GC-IPL in the macular region of patients with AD and aMCI and compared them to controls. The results showed that there were significant reductions in the thickness of the RNFL, GC-IPL, and macula of the severe AD group, as well as in the RNFL and macular thickness of the mild to moderate AD group, compared to the controls. Previous reports mostly conducted on the peripapillary region had demonstrated that AD patients showed a thinning of the RNFL [7, 8]. Our results confirmed these observations as well as suggested another important finding by adding macular ganglion cell analysis using the most recent technology, SD-OCT. Interestingly, histopathological studies have shown that the retinal ganglion cell loss in AD was found to be more extensive in the central retina than the peripheral area [2]. The GC-IPL thickness is a direct assessment of retinal ganglion cells in the macular area, whereas RNFL thickness is a measurement of ganglion cells axons. Taken together, the retinal degeneration observed in AD resulted in decreased peripapillary RNFL and macular thickness in our OCT study.

Recently, there has been a lot of interest in the relationship between neurodegenerative diseases and glaucoma. Previous reports have suggested that there is a link between AD, glaucoma, and abnormalities in the RNFL [17]. In that regard, the results of our study are reliable, since we excluded individuals diagnosed with glaucoma or increased intraocular pressure.

The clinical diagnosis of MCI has some limitations in distinguishing healthy patients with normal age-related forgetfulness, or patients with other neurodegenerative diseases, from those with dementia. That is, not every patient diagnosed with MCI progresses to AD. Consequently, there has been some controversy regarding previous reports of abnormalities in the RNFL in MCI patients. Among the subtypes of MCI, aMCI is considered a prodromal stage of AD [5]. There is emerging evidence that magnetic resonance imaging can observe neural deterioration, including progressive loss of gray matter in the brain, from aMCI to full-blown AD [18]. A technique known as Pittsburgh Compound B positron emission tomography imaging is used to clearly show the sites and shapes of beta amyloid deposits in live subjects using a ^11^C tracer that binds selectively to such deposits, which are frequently observed in aMCI and AD patients [19]. In addition, other radiotracers targeting not only beta amyloid, but also tau deposition, have been developed [20]. Recently, diagnostic criteria for the symptomatic predementia phase of AD were revised as clinical criteria, and research criteria incorporated the use of biomarkers based on imaging and cerebrospinal fluid measures [5].

Initially, we focused on aMCI and expected to show reduced retinal thickness in aMCI patients in this study. Unfortunately, our results had no significant difference between the aMCI and controls groups. Two previous studies evaluated peripapillary RNFL thickness and macular volume compared to controls, in which those results were different in aMCI patients. One showed that the overall RNFL was thinner and macular volume was increased in aMCI patients [11]. The other reported only temporal RNFL thinning and decreased macular volume in aMCI patients [12]. Since the RNFL and macular thickness decreased normally with age [21], age-matched controls are highly importance for analysis. In our study, the AD and control groups were the same age band without significant difference. However, the aMCI patients (68.6 ± 7.9 years) were younger than all the AD patients (75.0 ± 8.3 years, p = 0.017). Although we adjusted for the age difference in our statistical analysis, we failed to show any significant differences, likely due to our relatively small sample sizes. Hence, large studies with longitudinal data are required to investigate the change in retinal thickness over time to determine the use of OCT as a potential surrogate marker in the prognostication of those with aMCI.

The correlation between clinical severity of AD and retinal thickness is not well established. Although some studies report a significant association between MMSE and RNFL thickness [11], most articles found no significant correlation [9, 12]. The most crucial issue is that there is no proper indicator of severity of AD. For example, MMSE score is greatly influenced by age, education level, and medical and psychological conditions at the time of the exam. CDR and GDS are considered to allow more reliable staging of dementia than MMSE to assess overall dementia severity of AD patients [15]. Considering the neurodegenerative course of AD, retinal thickness might decrease with disease progression. As expected, we found a significant negative correlation between the clinical disease severity and retinal thickness.

Our study has several limitations. First, it assessed diagnosis and severity only by clinical criteria and by cognitive battery. In particular, clinical diagnosis for aMCI has the inevitable problem of subject inclusion because of those that develop dementia, about 30% do not meet the neuropathological criteria for AD [22]. Almost all of our aMCI patients had proceeded to dementia and the disease severity had progressed in the AD patients after we had observed our patients for several years. However, this study has similar or fewer limitations than previous clinical research. Second, our study only had a cross-sectional study design. Prospective and longitudinal studies are needed to verify the correlation between severity of AD and retinal thickness. Furthermore, if OCT measurements can be correlated with biomarkers such as beta amyloid or tau deposits, pathologic findings, or MRI data, more interesting and convincing data could be produced.

Although AD pathological changes can be detected in the cerebrospinal fluid, or using brain imaging with the development of diagnostic technology, the extent to which these changes contribute to the severity of AD is currently unknown [23]. For example, beta amyloid deposition is only weakly related to the degree of dementia. The known AD biomarkers are useful to differentiate between kinds of dementia, but not to assess disease severity. Therefore, this clinical approach is important for finding a putative biomarker to assess AD severity.

Although there have been many advancements in the diagnosis of aMCI and AD, tools for early diagnosis of these conditions are still lacking practically and would be of great use to neurologists. Our study shows that SD-OCT is a non-invasive tool which can detect retinal abnormalities in those with AD and may be a useful early marker of the disease.

## Supporting information

S1 File The raw data of the subjects. The demographics and OCT measurements of all the subjects
(PDF)
