# Supplementary material for: Decreased retinal thickness in patients with Alzheimer’s disease is correlated with disease severity
Source: PLoS One. 2019 Nov 5;14(11):e0224180. doi: 10.1371/journal.pone.0224180 (PMC6830808; doi:10.1371/journal.pone.0224180)
Supplement: S1 File — The demographics and OCT measurements of all the subjects (PDF) [file pone.0224180.s001.pdf]

number	Sex	Age	Group	MMSE	GDS	CDR	VOD	VOS	G.A.OD	G.M.OD	G.A.OS
1	M	71	s AD	18	5	1	0.9	0.9	69	65	70
2	M	81	m AD	19	3	1	0.9	0.9	61	49	61
3	M	72	m AD	23	4	1	0.8	0.6	96	74	70
4	M	53	m AD	25	4	1	1.0	1.0	.	.	.
5	F	74	s AD	15	5	1	.	.	82	80	76
6	F	74	s AD	8	6	2	0.5	0.5	64	59	64
7	F	78	s AD	12	6	.	.	.	46	23	.
8	F	82	s AD	9	5	1	0.1	0.2	.	.	.
9	F	82	m AD	11	4	1	0.6	0.7	.	.	92
10	M	65	s AD	3	6	3	.	1.0	.	.	76
11	F	71	m AD	16	5	1	1.0	1.0	69	58	67
12	M	79	m AD	20	4	1	0.7	0.9	73	63	74
13	M	83	s AD	10	5	1	0.2	0.2	56	0	74
14	M	87	m AD	14	4	1	0.5	0.5	69	50	76
15	F	70	s AD	18	5	1	0.7	0.8	72	63	54
16	F	58	aMCI	26	3	1	1.0	1.0	90	85	88
17	M	78	aMCI	26	3	1	0.6	0.5	78	67	37
18	F	71	aMCI	21	3	1	0.6	0.5	86	80	88
19	F	67	aMCI	23	3	1	1.0	1.0	73	65	74
20	F	52	aMCI	26	3	1	1.0	1.0	90	86	90
21	F	70	aMCI	26	3	1	.	.	.	.	.
22	F	77	aMCI	23	3	1	.	.	.	.	.
23	M	78	aMCI	25	3	1	1.0	0.9	72	69	74
24	M	75	aMCI	24	3	1	1.0	1.0	82	76	81
25	F	78	m AD	17	4	1	1.0	1.0	74	63	76
26	M	71	aMCI	24	3	1	0.4	0.9	69	67	68
27	M	67	aMCI	25	3	1	1.2	1.2	87	85	84
28	F	70	aMCI	21	3	1	1.0	0.9	90	88	93
29	M	58	aMCI	27	3	1	1.2	1.2	93	90	93
30	F	69	aMCI	22	3	1	0.3	1.0	34	.	82
31	M	69	control	.	.	.	0.8	0.6	80	76	80
32	M	67	control	.	.	.	0.7	0.7	72	70	67
33	M	63	control	.	.	.	1.0	0.9	79	73	89
34	F	64	control	.	.	.	0.9	1.0	93	84	92
35	F	73	control	.	.	.	0.7	1.0	77	74	77
36	F	68	control	.	.	.	0.7	0.7	42	11	78
37	F	71	control	.	.	.	0.8	0.8	70	63	72
38	M	64	control	.	.	.	1.0	0.9	75	56	86
40	F	63	control	.	.	.	1.0	1.0	83	83	83
43	F	63	control	.	.	.	1.0	1.0	74	70	75
44	F	65	control	.	.	.	1.2	1.2	84	80	83
45	M	64	control	.	.	.	1.0	1.2	83	80	94

48	M	76	control	.	.	.	0.8	1.0	73	69	70
49	F	73	control	.	.	.	0.8	0.7	80	74	77
51	F	76	control	.	.	.	1.0	1.0	80	62	81
52	F	63	control	.	.	.	0.9	0.9	79	77	81
55	M	66	control	.	.	.	1.0	1.0	85	82	85

G.M.OD_A	M.OCTOD	M.OCTOS	R.OCTOD	R.OCTOS	R.OCTD.S	R.OCTD.I	R.OCTD.N
67	240	242	81	79	107	117	53
58	263	270	46	54	66	56	16
46	261	247	92	98	109	139	60
.	.	.	88	89	129	114	47
43	468	315	95	102	108	121	70
64	222	215	65	70	82	78	53
.	239	237	66	72	63	91	57
.	241	248	56	59	63	66	55
77	253	244	79	96	75	117	66
75	.	224	.	.	.	.	.
60	258	256	84	81	119	113	62
65	262	263	82	89	94	99	68
67	.	.	88	89	109	102	69
65	269	272	81	67	98	104	62
27	263	236	78	68	86	108	56
84	215	214	92	93	121	128	61
13	242	186	89	79	107	113	65
69	236	245	96	90	99	129	79
70	252	248	82	81	94	130	57
83	251	250	104	104	135	128	70
.	.	.	91	90	117	127	61
.	.	.	79	89	106	60	55
73	272	278	76	78	96	96	52
75	282	278	94	92	119	115	80
72	281	284	83	86	88	107	63
64	269	267	90	85	116	119	60
49	285	279	88	85	104	99	73
89	316	315	101	100	127	134	77
91	286	286	96	100	117	124	75
76	230	246	97	93	146	129	60
75	230	228	98	101	141	132	68
65	259	261	90	85	113	111	76
87	256	264	85	107	105	120	52
89	262	251	102	108	130	99	92
74	245	256	78	83	94	100	54
66	267	245	83	93	88	115	67
65	257	254	100	99	118	163	69
81	248	252	79	89	69	127	73
82	246	242	93	97	103	111	68
72	267	261	86	81	102	125	66
82	279	252	88	83	89	136	58
91	285	291	87	92	115	108	66

66	246	243	84	78	113	107	58
57	280	282	86	89	108	113	65
78	274	269	90	97	116	119	55
78	272	274	89	87	120	108	69
82	283	279	96	98	118	131	61

R.OCTD.T	R.OCTS.S	R.OCTS.I	R.OCTS.N	R.OCTS.T
47	103	107	61	47
47	65	61	49	41
59	124	130	67	72
64	122	117	66	53
81	133	120	66	90
48	84	85	51	59
52	86	90	50	62
40	64	64	69	37
57	129	116	58	79
.	.	.	.	.
41	92	116	71	45
60	101	115	68	73
72	110	106	62	74
62	104	59	50	57
63	90	88	55	39
60	129	129	59	56
72	100	89	65	62
77	97	125	75	62
47	103	121	56	45
82	130	134	81	70
59	109	135	63	54
93	97	128	55	78
60	94	97	64	58
62	134	90	81	64
72	107	102	72	64
66	104	110	71	54
76	86	106	61	77
68	136	125	66	73
67	129	136	62	73
54	133	121	59	58
53	141	134	72	58
59	106	107	73	55
63	144	136	76	73
86	118	154	85	76
63	107	110	48	67
62	119	126	63	64
47	127	155	66	47
48	96	129	67	63
92	105	121	71	89
53	103	109	58	53
67	95	118	61	58
59	121	120	65	63

60	100	101	57	55
59	113	117	61	63
72	110	129	54	56
60	116	116	56	58
76	148	118	55	72
